# Dual-modal magnetic resonance and photoacoustic tracking and outcome of transplanted tendon stem cells in the rat rotator cuff injury model

**DOI:** 10.1038/s41598-020-69214-5

**Published:** 2020-08-18

**Authors:** Xueqing Cheng, Jinshun Xu, Ziyue Hu, Jingzhen Jiang, Zhigang Wang, Man Lu

**Affiliations:** 1 Ultrasound Medical Center, Sichuan Cancer Hospital & Institute, Sichuan Cancer Center, Cancer Hospital Affiliated to School of Medicine, University of Electronic Science and Technology of China, Chengdu, 610041 China; 2 Department of Ultrasound, West China School of Medicine, West China Hospital, Sichuan University, Chengdu, 610041 China; 3 North Sichuan Medical College, Nanchong, 637100 China; 4 Second Affiliated Hospital of Chongqing Medical University & Chongqing Key Laboratory of Ultrasound Molecular Imaging, Chongqing, 400010 China

**Keywords:** Stem-cell research, Molecular medicine

## Abstract

Stem cells have been used to promote the repair of rotator cuff injury, but their fate after transplantation is not clear. Therefore, contrast agents with good biocompatibility for labeling cell and a reliable technique to track cell are necessary. Here, we developed a micron-sized PLGA/IO MPs to label tendon stem cells (TSCs) and demonstrated that PLGA/IO MPs were safe and efficient for long-term tracking of TSCs by using dual-modal MR and Photoacoustic (PA) imaging both in vitro and in rat rotator cuff injury. Moreover, TSCs improved the repair of injury and the therapeutic effect was not affected by PLGA/IO MPs labeling. We concluded that PLGA/IO particle was a promising dual-modal MR/PA contrast for noninvasive long-term stem cell tracking.

## Introduction

Rotator cuff injury is one of the most common shoulder diseases, which occur most often in people who repeatedly perform overhead motions in their jobs or sports^1^. The conventional therapies used for rotator cuff injury include analgesics, anti-inflammatory drugs, physiotherapy, steroids injection, and surgical repair^2^. In fact, conservative treatments usually show short-term pain relief but lack long-term efficacy^3^. Despite advances in surgical treatment options, the failure rates of rotator cuff repairs were still as high as 20%^4^. In recent years, stem-cell-driven regeneration is gaining increased attention, and mesenchymal stromal cells (MSCs) have been widely applied for the treatment of rotator cuff tears on the basis of their self-renewal, clonogenicity, and multidifferentiation potential^5–12^. But Mohammad et al. found that intra-synovial implantation of marrow-derived MSCs did not promote tendon healing in ovine deep digital flexor tendon injury model^12^.

While, tendon stem cells (TSCs) display high clonogenecity, cell proliferation, and tenogenic-differentiation potential compared to bone marrow mesenchymal stromal cells since Bi et.al firstly identified them in 2007, suggesting that they could be a better cell source for tendon regeneration^13^. To develop effective TSCs therapies, it is imperative to label and track the administrated cells, preferably in a noninvasive manner with high efficiency.

Recently, optical imaging, radionuclide imaging, magnetic resonance imaging (MRI), ultrasound (US), photoacoustic (PA) imaging and computed tomography (CT), have been widely used for cell tracking^14–19^. However, each individual imaging has its own advantages and limitations. Optical imaging suffers from shallow penetration depth and phototoxicity, and radionuclide imaging has poor spatial resolution and rapid decay of radioisotopes^20,21^. MRI, as the gold standard stem cell tracking modality, has high spatial resolution and deep penetration but has a relatively poor temporal resolution^19,22^. While, PA have excellent temporal resolution but suffer from poor penetration depth^16,23^. Therefore, nanoparticles based dual-/multi-modal imaging modalities that allow long-term tracking of stem cells with nontoxicity and good biocompatibility are need^24,25^.

**Figure 1:**
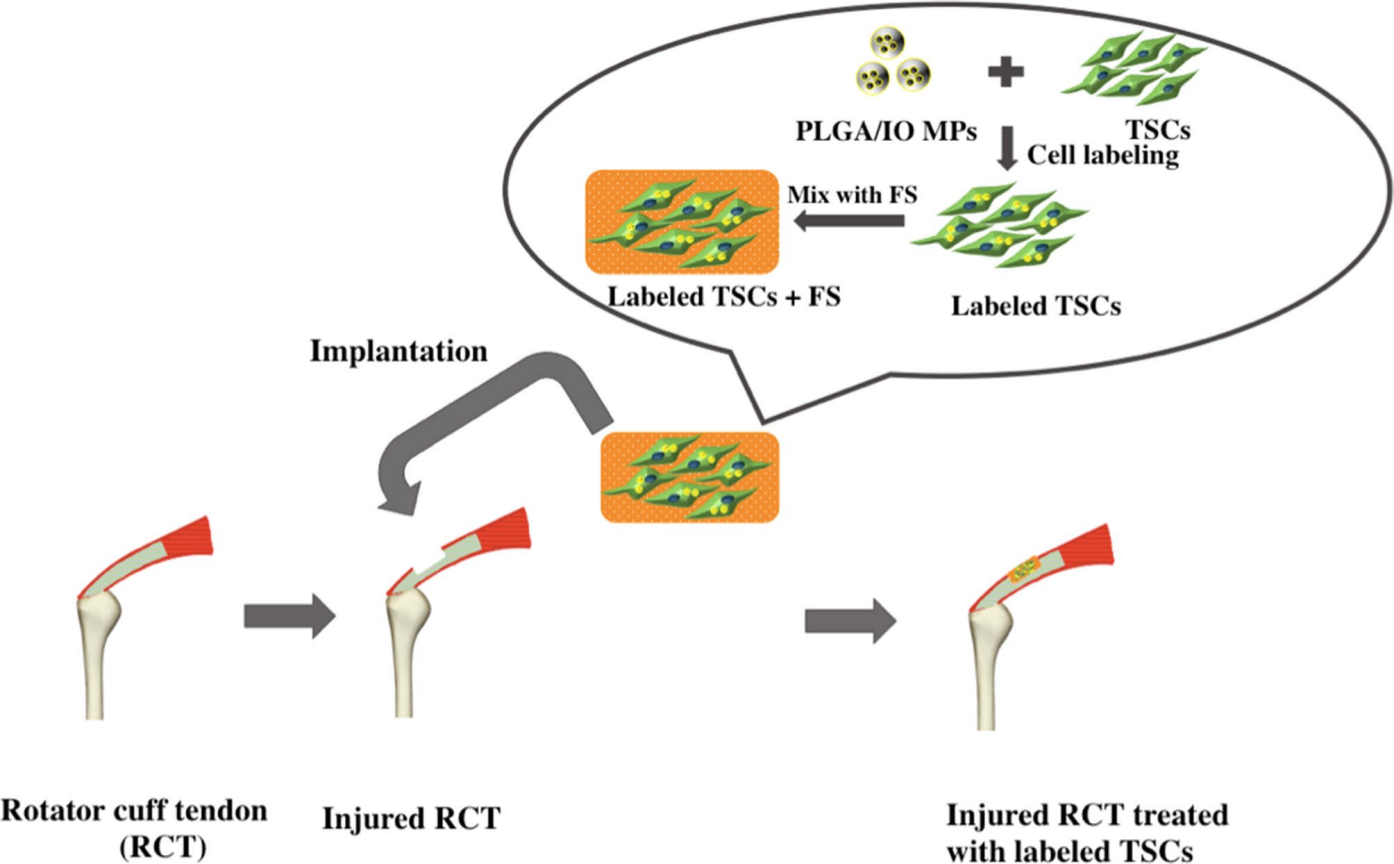
Diagram of the procedure for implantation of tendon stem cells (TSCs) into a rat rotator cuff tendon injury model. *FS* fibrin sealant.

In this study, a micron-sized Poly lactic-co-glycolic acid particles embedded with iron oxide nanoparticles (PLGA/IO MPs) was fabricated, and TSCs are labeled with PLGA/IO MPs in vitro and then implanted in the rat rotator cuff injury model (Fig. 1). We aimed to detect the efficiency of PLGA/IO MPs for long-term tracking of TSCs by using dual-modal MRI and PA imaging. Additionally, whether the outcome of TSCs transplantation is affected by PLGA/IO MPs labeling was determined in a rat rotator cuff injury model. To our knowledge, this is the first study of dual-modal MR/PA tracking of TSCs by using a single iron oxide nanoparticle as contrast agent in a rat rotator cuff injury model.

## Methods

### Labeling TSCs with PLGA/IO MPs-PLL

Both PLGA/IO MPs and TSCs were prepared using the same procedure with our previous study^25^, as described in detail in Supplementary Materials. TSCs were grown in DMEM (Gibco), supplemented with 10% (v/v) fetal bovine serum (Hyclone) and 1% (v/v) penicillin/streptomycin (Beyotime) at 37 °C, 5% CO_2_.

For high loading of cells with particles, we incubated the PLGA/IO MPs with the cationic transfection agent Poly-l-lysine (0.01%) for 40 min at room temperature to make particles positively charged. The positively charged particles make a stronger electrostatic interaction with negatively charged cell membrane^22^. At 80–85% confluency, TSCs were labeled with 100 μg Fe/mL of PLGA/IO MPs-PLL in fresh culture medium for 12 h^25^. After washing with sterile PBS three times to remove all free particles, labeled TSCs were trypsinized using 0.25% trypsin/EDTA. Detached cells were collected by centrifugation at 1,000 rpm/min for 5 min, resuspended, and counted by hemocytometer. The P3–P7 TSCs were used for all experiments.

TSCs were labeled with the fluorescent PLGA/IO MPs (incorporating DiI during the preparation of particles) to facilitate the identification of particles internalized within TSCs^25^. At 4 h after labeling, TSCs were fixed with 4% paraformaldehyde, and then stained with fluorescent dyes (with DiO staining plasma membrane and DAPI staining nucleus) and Prussian blue (Beijing Leagene Biotech. Co., Ltd.) according to the manufacturer’s respectively instructions.

CCK-8 assay (Cell Counting Kit-8 reagent, Dojindo, Lot.JM754) was performed in triplicate to detect the viability of TSCs. Labeled TSCs (5 × 10^3^ cells per well) were seeded into a 96-well cell culture plate at 37 °C, 5% CO_2_ for 24 h. Different concentration of PLGA/IO MPs-PLL (0, 25, 50, 100 and 200 μg Fe/mL) were added and incubated for 24 h at 37 °C. Cells were then washed with PBS followed by treatment with 100 μL of culture media and 20 μL of assay reagent in each well. The samples were incubated for 2 h, and the absorbance at 490 nm was then recorded using a microplate reader (Victor3, PerkinElmer, Waltham, MA, USA).

### Iron quantification in TSCs

At 3, 7, 14, 21, and 28 days, labeled TSCs were collected and assayed to quantify iron contents using inductively coupled plasma optical emission spectrometry (ICP-OES). 0.1 mL of labeled TSCs suspension was digested overnight using 0.4 mL 70% concentrated nitric acid. Samples were then diluted to a volume of 10 mL with deionized water, yielding a final nitric acid concentration of 2%^22^. An iron standard (Catal. #43149, Sigma-Aldrich) was used to obtain the standard curve and iron concentration was determined.

### In vitro MR/PA imaging

Agarose was added to distilled water at 1% and heated at 60 °C until use. The agarose solution (500 μL) was then mixed with labeled TSCs solutions (500 μL) in a 1.5 mL EP tubes. Then, 100 μL heated sample were pipetted from the EP tube and added into a 3% agarose gel model with holes for PA imaging. Samples (900 μL) in the EP tube were allowed to set for MR imaging. For the first set of experiments, different numbers of labeled TSCs (5 × 10^6^, 1 × 10^6^, 5 × 10^5^, 1 × 10^5^, 5 × 10^4^ and 1 × 10^4^ cells) were suspended in 500 μL of PBS and mixed with 500 μL of 1% agarose in PBS. The final cell concentrations were 5 × 10^6^, 1 × 10^6^, 5 × 10^5^, 1 × 10^5^, 5 × 10^4^ and 1 × 10^4^ cells/mL in 0.5% agarose, respectively. A control was made with 5 × 10^6^ cells/mL unlabeled TSCs in 0.5% agarose solution.

PA imaging was performed using a Visual Sonics Vevo LAZR-2100 high-frequency photoacoustic system (Canada), a 20 MHz array transducer and pulsed laser excitation at 700 nm was applied for capturing PA signal. MRI was performed using a 3.0 T MRI (Philips Chielva) with a head coil using the T2* sequence (TR = 232 ms, TE = 9.21 ms, 8 echo, FOV = 180 mm, matrix = 128 × 128, slice thickness = 3 mm, flip angle 45°). Both MR and PA signal value of TSCs were measured by drawing the outline of the ROI.

Next, 1 × 10^6^ cells/mL labeled were collected for MR and PA imaging at day 3, 7, 14, 21 and 28 after labeling respectively using the same methods.

### TSCs implantation on a rat rotator cuff injury model

To evaluate the effect of TSCs implantation on tendon repair, we established a rat rotator cuff injury model. All animal experiments were approved by our animal ethics committee, and all experiments were performed in accordance with relevant guidelines and regulations. The process diagram was shown in Fig. 1. Briefly, the skin and subcutaneous tissue was incised on the anterolateral aspect of the shoulder, and then the deltoid was split to reveal the supraspinatus tendon (Fig. S1). A no. 11 scalpel blade (Jinhuan Healthcare, Shanghai, China) was used to scratch the surface of supraspinatus tendon repeatedly, creating a partial-thickness rotator cuff defect (3 mm × 3 mm) at a distance of 3 mm from its insertion on the greater tuberosity. The procedure was performed in bilateral rotator cuff tendon among 42 SD rats, the right was randomized to receive either 10^6^ labeled TSCs in a fibrin carrier (labeled TSCs group, n = 21) or 10^6^ unlabeled TSCs in a fibrin carrier (unlabeled TSCs group, n = 21), and the left was randomized to receive either the fibrin carrier alone (FS group, n = 21) or to receive no implant (untreated group, n = 21).

The fibrin sealant product (FIBINGLURAAS, Shanghai RAAS Blood Products Co., Ltd., Shanghai, China) was used for cell delivery. Thrombin was dissolved in calcium chloride solution to yield thrombin solution in one syringe. Fibrinogen was then dissolved in sterile water for injection in a 37 °C to yield fibrinogen solution in another syringe. The fibrin sealant product instantaneously formed a gel when the solutions from each syringe were mixed. Firstly, the cell suspension (labeled or unlabeled TSCs) was mixed with the thrombin solution at a 1:1 ratio, and then the cell-thrombin suspension was mixed with the fibrinogen solution at a 1:1 ratio using the Disposal Connected Mixing System (Shanghai RAAS Blood Products Co., Ltd.) and simultaneously added to each well on the surface of the injured tendon^5,11,26^.

Finally, skin was sutured with 4-0 Vicryl sutures. Post-operative rats were maintained in cages and allowed to move without restriction. All animal experiments were approved by the Animal Ethics Committee of Chongqing Medical University. 2% pentobarbital sodium salt solution (2 mL/kg) was intraperitoneal injected to anesthetize rats.

### In vivo MR/PA imaging

To track implanted TSCs via MR and PA imaging, right rotator cuff that received labeled TSCs was examined using both MRI and PA imaging at day 3, 7, 14, 21 and 28 after implantation respectively. MRI studies were conducted in a 7.0 T horizontal bore small animal MRI scanner (Bruker Biospin). All rats were anesthetized with 1–2% isoflurane mixed with pure oxygen via a nose cone and were placed in a lateral position with the examined shoulder side up with a respiratory sensor. Axial, sagittal, and coronal two-dimensional (2D), fast spin echo sequence images were first obtained to ensure the imaging position of the rotator cuff. T2-weighted multi-slice spin echo images were acquired in coronal plane of rat shoulder (TR/TE = 3,000/30 ms, matrix = 256 × 256, FA = 30, 28 contiguous slices).

PA images were acquired by using the Visual Sonics Vevo LAZR-2100 high-frequency photoacoustic system. All rats were anaesthetized by 2% pentobarbital sodium salt solution. The hairs around the interested shoulder were shaved before PA imaging. Firstly, B-mode was used to locate the site of rotator cuff injury. Then PA-mode was switched on for capturing PA signal. The combined ultrasound and photoacoustic (US/PA) images were obtained and stored for image analysis.

### Tissue preparation and histological analysis

After 3, 7, 14, 21 and 28 days, the supraspinatus tendon (n = 3/time point/group) was harvested and processed for histological analysis. The specimen was fixed in 10% buffered formalin overnight, washed, and dehydrated through a graded series of alcohol and embedded in paraffin. Sections 4-mm thick were acquired from the tendon specimens and then stained with hematoxylin and eosin (H&E), Masson’s trichrome and Prussian blue staining according to manufacturer instructions. H&E staining was employed to assess overall morphology, and masson’s trichrome staining was used to distinguish tendon connective tissue from muscle and bone. While Prussian blue staining was performed to detect blue-stained iron particles in tissue.

### Tensile testing apparatus and testing procedure

Rats were sacrificed at 8 weeks after implantation to harvest the supraspinatus tendon including the humeral bone and supraspinatus muscle. Biomechanical testing of the supraspinatus tendons (n = 6/group) was performed using an electrodynamic test machine (BOSE Electroforce 3330, USA). And specimen (n = 6) was also obtained from three healthy rats and used for comparisons with the untreated group, labeled TSCs group, unlabeled TSCs group and FS group. Each tendon was stretched at a rate of 0.5 mm/s until failure. The supraspinatus tendon was fixed to this system and loaded until it ruptured at its midsubstance (Fig. S2). The ultimate load to failure was recorded for later statistical analysis.

### Statistical analysis

Measurement data were shown as mean along with standard deviation, and the statistical significance were determined by Student’s *t* test or one- way ANOVA followed by Tukey’s multiple comparison using GraphPad Prism software. *P* values less than 0.05 were considered statistically significant.

## Results

### Characterization of PLGA/IO MPs (PLL) and labeled TSCs

**Figure 2:**
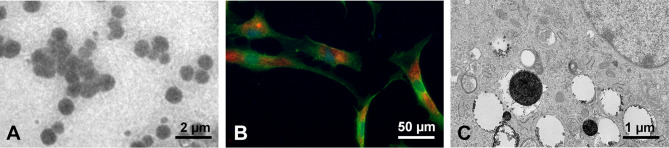
Characterization of PLGA/IO MPs. (**A**) TEM image of PLGA/IO MPs. (**B**) Fluorescent image of TSCs after labeling with 100 μg Fe/mL PLGA/IO MPs. (**C**) TEM image showed that PLGA/IO MPs (arrows) were internalized in a TSC. Please note that **C** has been ^25^ reproduced from our previous article to show the internalisation of the PLGA/IO MPs in TSCs by TEM.

The average particle size of PLGA/IO MPs was 974.6 ± 146.1 nm and the zeta potential was − 12 ± 3.88 mV (Fig. S3A,B). And the coating of PLGA/IO MPs with PLL led to a surface charge of 13 ± 5.2 mV (Fig. S3C). Transmission electron microscope images (TEM) showed that PLGA/IO MPs was spherical in shape, and IO-NPs were encapsulated within the core of PLGA-MPs (Fig. 2A). TSCs are directly labeled with these positive charged particles by co-incubation in a culture medium. The internalization of PLGA/IO MPs into rat TSCs was shown by fluorescent image and TEM (Fig. 2B,C). The particles were observed to accumulate in cytoplasm vesicles of TSCs^25^.

### MR/PA tracking of PLGA/IO MPs labeled TSCs in vitro

**Figure 3:**
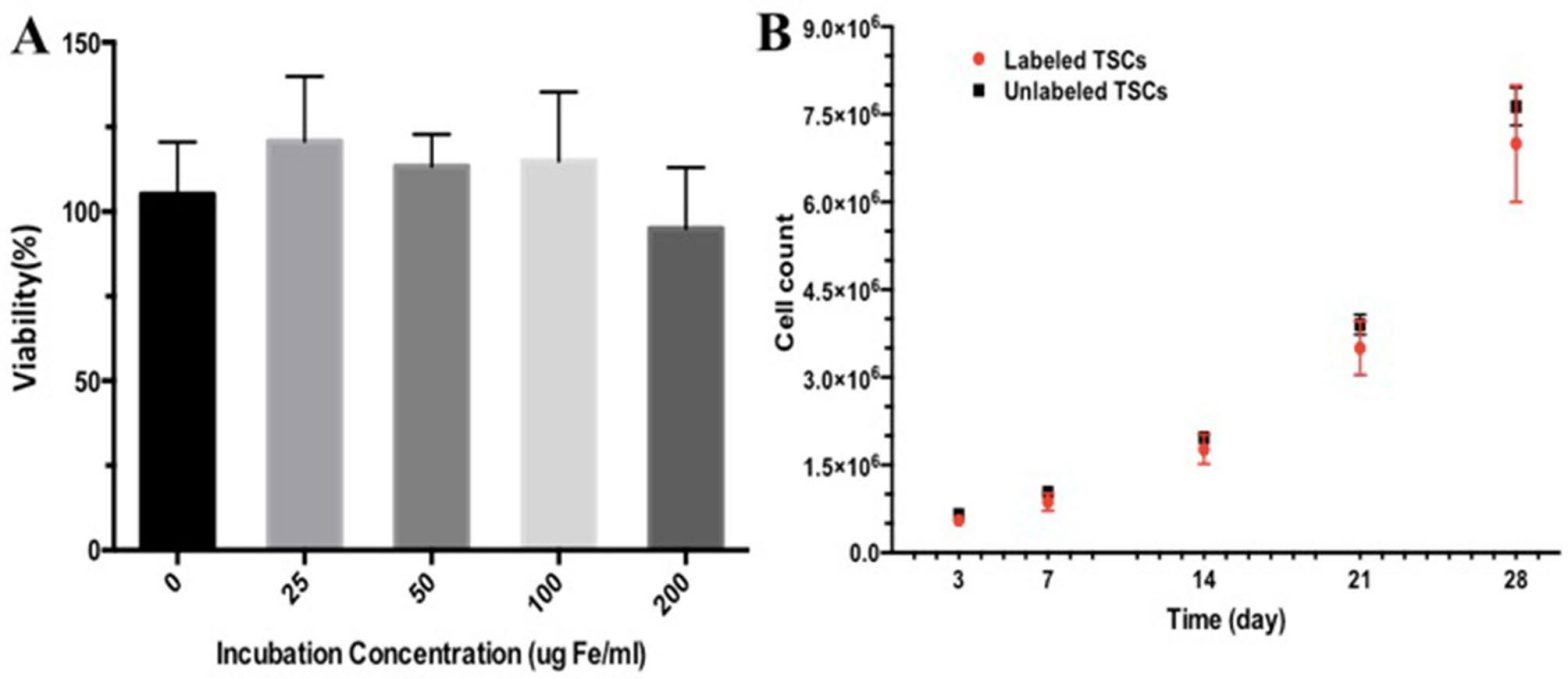
Impact of particle labeling on cell viability and proliferation. **A** Viability of PLGA/IO MPs TSCs as a function of iron concentration during the incubation. **B** Proliferation of TSCs labeled with PLGA/IO MPs.

To exclude the possible adverse effect of PLGA/IO MPs labeling on cell viability, the cytotoxicity of PLGA/IO MPs was examined. The CCK8 assay showed no cytotoxic effects of the particles even at a high iron concentration (up to 200 μg Fe/mL). No significant differences in viability were seen between unlabeled and labeled cells (*P* > 0.05, two-tailed *t* test) (Fig. 3A). Furthermore, there was no difference in the growth rate between labeled TSCs and unlabeled TSCs with our protocol (incubation with 100 μg Fe/mL PLGA/IO MPs for 12 h) (Fig. 3B).

**Figure 4:**
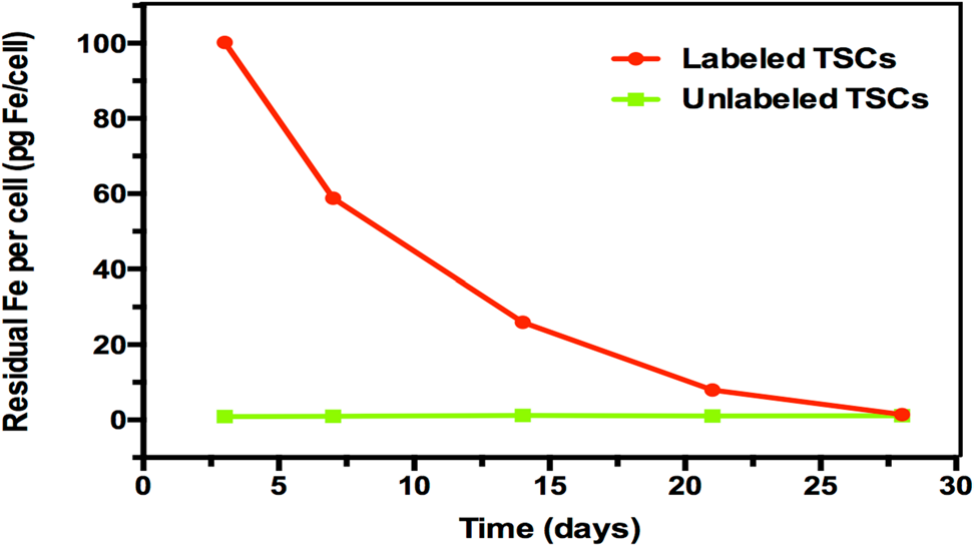
Change in cellular iron content per cell after initial labeling with 100 μg Fe/mL PLGA/IO MPs.

ICP-OES quantification revealed the amount of Fe loaded into the cells was gradually decreased over time. The Fe loading/ cell was 100.2 pg Fe/cell at day 3, and decreased to half of its initial value at 7 days. At day 28, the iron concentration per cell was close to background (1.45 pg Fe/cell VS 1.17 pg Fe/cell, *P* > 0.05) that was undetectable neither by PA nor by MR imaging (Fig. 4E). It revealed the potential of PLGA/IO MPs for long-term labeling of TSCs in vitro.

**Figure 5:**
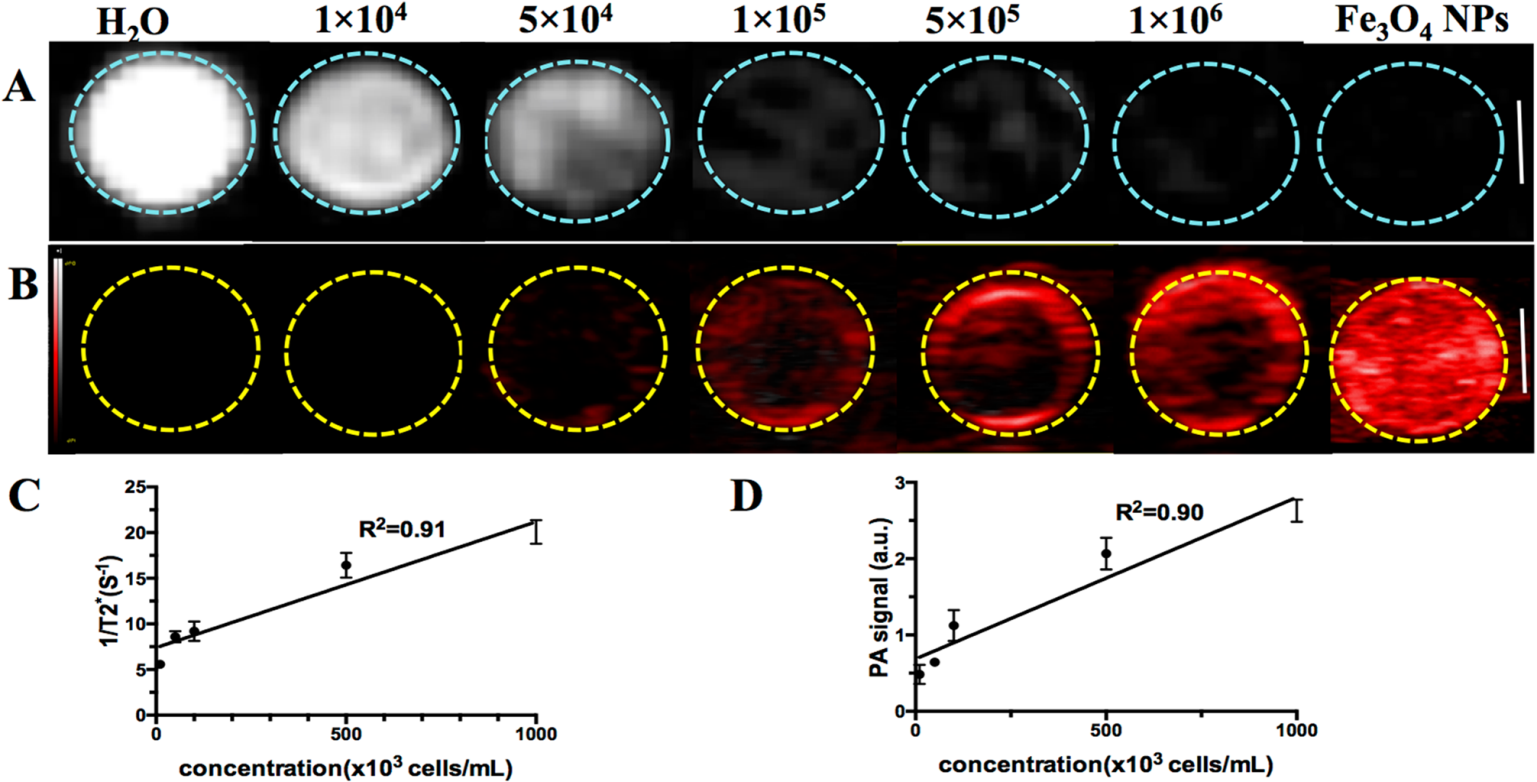
MR and PA imaging of different concentration (1 × 10^4^, 5 × 10^4^, 1 × 10^5^, 5 × 10^5^, 1 × 10^6^ Cells/mL) of PLGA/IO MPs labeled TSCs. **A** T2^*^ images of labeled TSCs in agarose gel (blue dotted circle). Scale bar = 5 mm. **B** PA images of labeled TSCs in agarose gel (red dotted circle). Scale bar = 2 mm. **C** The quantitative analysis of MR signal (1/T2^*^). **D** The quantitative analysis of PA signal. Iron oxide nanoparticles (Fe_3_O_4_ NPs; 100 μg/mL) is a positive control for both MR and PA; water (H_2_O) is a negative control.

The previous study indicated that the PLAG/IO MPs labeled TSCs could be detected by both MRI and PA imaging^25^. To examine the detecting limit by MRI and PA imaging, different amounts of labeled TSCs mixed in equal agarose gel were imaged. As shown in T2-weighed MR images and PA images (Fig. 5), the negative MR signal (1/T2) and positive PA signal of labeled TSCs increased with the increase of TSCs concentration (R^2^ = 0.9, R^2^ = 0.91 respectively). And no signal could be detected in the untreated TSCs. This result indicated that an enhanced MR and PA signal could be detected for labeled TSCs as low as 5 × 10^4^ cells/mL, 1 × 10^5^ cells/mL respectively in vitro.

**Figure 6:**
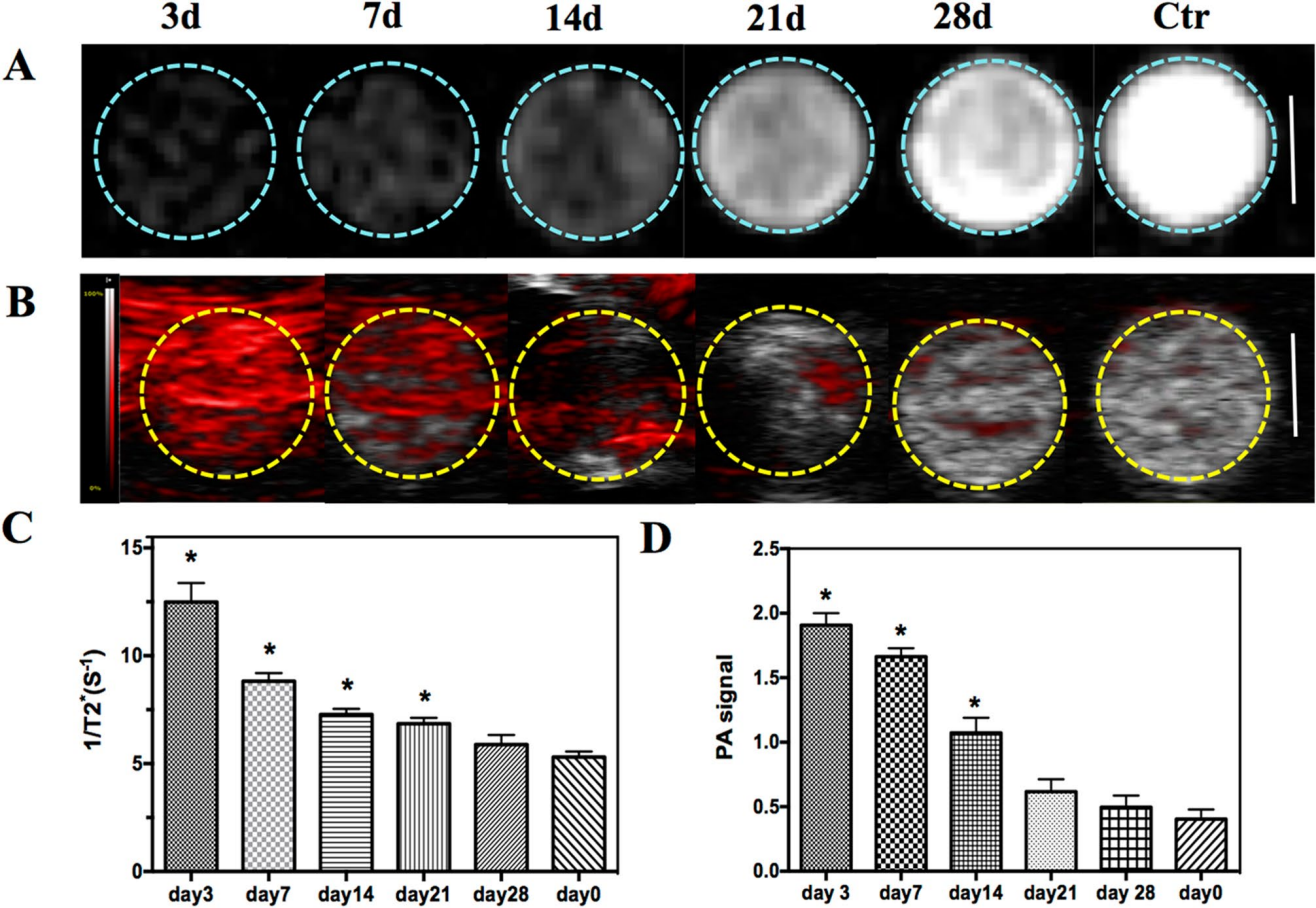
Efficiency of PLGA/IO MPs for long-term tracking of TSCs in vitro. **A** T2^*^-weighted MR images of labeled TSCs at different time point and unlabeled TSCs (Ctr) in agarose gel. Scale bar = 5 mm. **B** PA images of labeled TSCs at different time point and unlabeled TSCs (Ctr) in agarose gel. Scale bar = 2 mm. **C** The intensity of PA signal value of labeled TSCs at different iron concentrations by comparison with unlabeled TSCs (Day 0). **D** The intensity of MR signal value of labeled TSCs at different iron concentrations by comparison with unlabeled TSCs (Day 0). ^*^, *P* < 0.05.

To explore the efficiency of PLGA/IO MPs for longitudinal labeling of TSCs in vitro, 1 × 10^6^ cells/ml labeled TSCs were dispersed in 0.5% agarose gel and imaged by MR/PA imaging at 3, 7, 14, 21 and 28 days after labeling. As shown in Fig. 6A, labeled TSCs showed as prominent hypointensity at day 3, then gradually decreased over time, and became invisibly detected at day 28. While, labeled TSCs showed enhanced PA signal on PA images at day 3, 7 and 14 except at day 21 and 28 as outlined in yellow (Fig. 6B). The quantitatively analysis of MR and PA signal of labeled TSCs in holes (Fig. 6C,D) demonstrated that both MR signal value (1/T2*) and PA signal of labeled TSCs were gradually decreased, and nearly decreased to the level of unlabeled TSCs at day 28 and day 21 respectively (*P* > 0.05). It may indicate that MR and PA imaging allow a long-term tracking of labeled TSCs for at least 21 days and 14 days in vitro respectively.

### MR/PA tracking of PLGA/IO MPs labeled TSCs in the rat rotator cuff injury model

**Figure 7:**
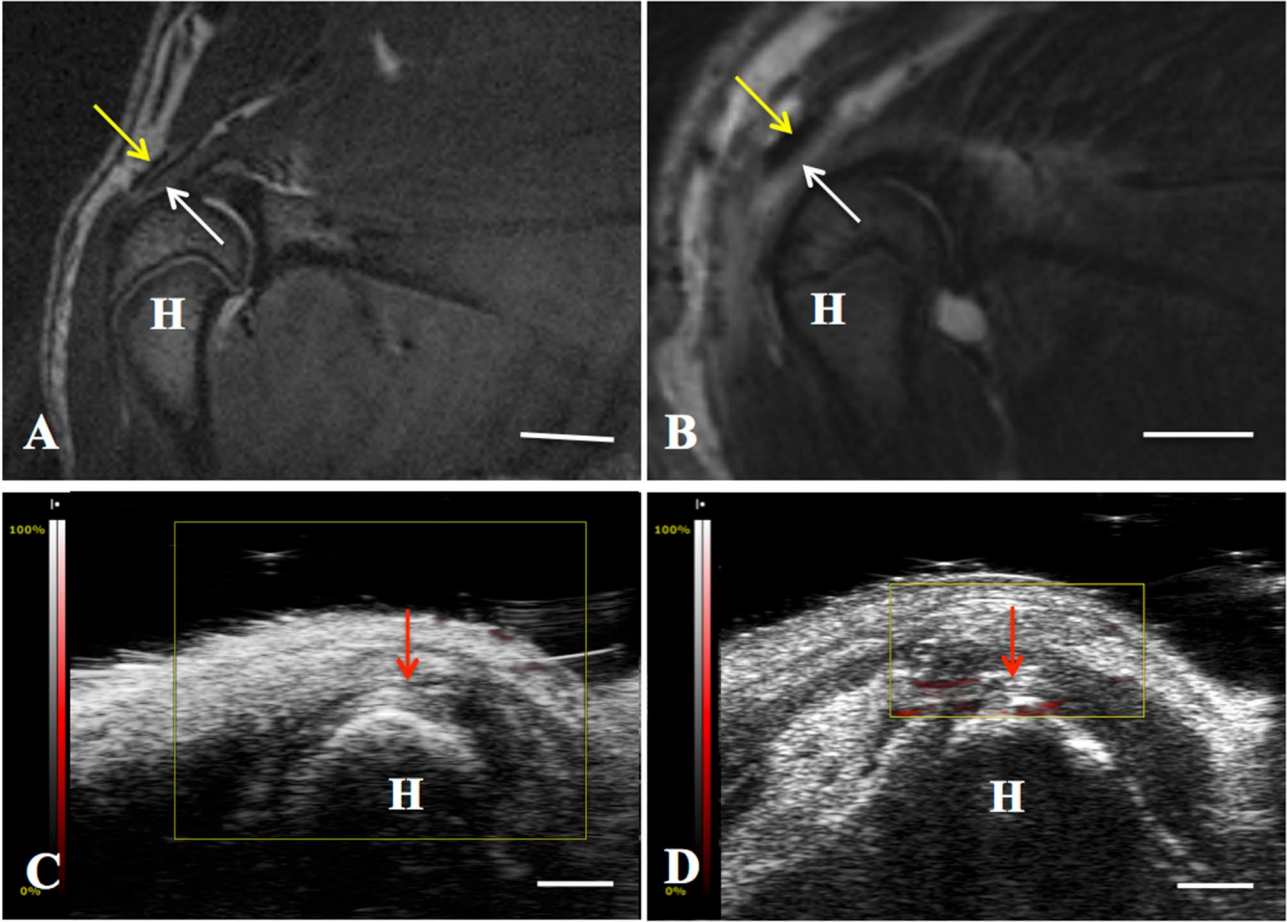
MR and PA images of rat rotator cuff tendon (the supraspinatus tendon). **A** Normal tendon (white arrow) manifested as low to intermediate signal under the acromion (yellow arrow) on T2-weighed sequence. Scale bar = 5 mm. **B** Injured tendon at 3 days after injury manifested as high signal on T2-weighed sequence. Scale bar = 5 mm. **C** Normal tendon (red arrow) on US/PA image. Scale bar = 2 mm. **D** Injured tendon (red arrow) at 3 days on US/PA image. *H* humeral head. Yellow arrows indicated the acromion of rat. Scale bar = 2 mm.

**Figure 8:**
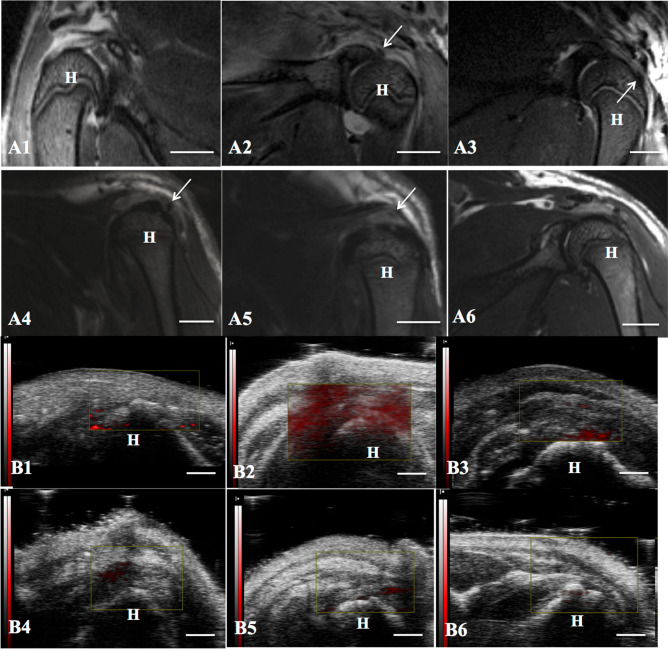
Efficiency of PLGA/IO particles for long-term tracking of TSCs in the Rat Rotator Cuff Injury Model. **A** MR images of injured rat rotator cuff treated with unlabeled TSCs (**A1**) and labeled TSCs at 3 (**A2**), 7 (**A3**), 14 (**A4**), 21 (**A5**) and 28 (**A6**) days after implantation. There was no hypointensity within the injured tendon in the unlabeled TSCs group (**A1**), while well-defined hypointensity (“black spots”, arrow) were observed at the region of cell implantation in the labeled TSCs group (**A2**) at 3 days after implantation. The hypointensity signal shrank and faded gradually at day 7 (**A3**), 14 (**A4**), 21 (**A5**), and became nearly invisible at day 28 (**A6**). Scale bars = 5 mm. (**B**) PA images of injured rat rotator cuff treated with unlabeled TSCs (**B1**) and labeled TSCs at 3 (**B2**), 7 (**B3**), 14 (**B4**), 21 (**B5**) and 28 (**B6**) days after implantation. There was little red signal around the tendon in the unlabeled TSCs group (**B1**), and diffusely positive signal (red) was detected within tendon in the labeled TSCs group (**B2**) at day 3. The red signal region shrank at day 7 (**B3**), shaded and became nearly undistinguishable from the background tissue at day 14 (**B4**), 21 (**B5**) and 28 (**B6**). Arrows indicated the hypointensity within the injured tendon. *H* humeral head. Scale bars = 2 mm.

We made an acute partial-thickness rotator cuff tendon tear model in rats to detect the efficiency of dual-modal MR/PA tracking of TSCs in vivo. As shown in Fig. 7A, the intact rotator cuff accurately presented as low to intermediate MR signal on T2-weighted sequence acquired by a 7.0 T MR scanner, while the injured rotator cuff showed as hyperintensity due to fiber disruption and edema caused by surgical procedures (Fig. 7B). It produced a favorable contrast for detection and tracking of PLGA/IO MPs labeled TSCs (shown as hypointensity) in the rat rotator cuff injury model. Well-defined hypointensity (“black spots”) were observed at the region of cell implantation in the labeled TSCs group (Fig. 8A2). While rats in the unlabeled TSCs group had no hypointensity on the tissue MRI appearance (Fig. 8A1). In coronal plane T2-weighted images of rat rotator cuff, spherical or coalescing regions of hypointensity were present most conspicuously near the site of implantation on both day 3 and 7 (Fig. 8A2,A3), and developed into dispersed and smaller foci of hypointensity at day 14 (Fig. 8A4). The hypointensity could still be visualized by MRI at day 21 (Fig. 8A5), but became nearly indiscernible from the surrounding tissue at day 28 (Fig. 8A6). This may indicate that the implantation of 1 × 10^6^ labeled TSCs embedded with fibrin sealant were detectable from a bright background of the injured rotator cuff tendon for up to three weeks by 7.0 T MRI.

For PA scanning, the combined US/PA images showed both the structural information and the PA signal of rat rotator cuff. We didn’t detect enhanced PA signal around the normal rotator cuff (Fig. 7C). And there was only a little PA signal around the injured rotator cuff (Fig. 7D). PA imaging clearly detected subjectively greatest quantity of the nanotracer signal (red) near the site of implantation in the labeled TSCs group at day 3 (Fig. 8B2). Then the diffuse red signal region throughout the tendon became shrank at day 7 (Fig. 8B3), shaded and became nearly undistinguishable from the background tissue at day 14, 21 and 28 (Fig. 8B4–B6). However, US/PA images showed little red signal around the tendon in the unlabeled TSCs group (Fig. 8B1).

Finally, the Prussian blue staining of histological sections showed that the distribution of blue-staining iron particles was only detected in the labeled TSCs group (Fig. S4). And it decreased gradually on tissue sections over time from day 7 to day 28 (Fig. S5). The distribution of particles in MR images correlated well to corresponding histological sections stained with Prussian blue, confirming that the hypointense spot originated from PLGA/IO MPs as opposed to other phenomena, such as hemorrhage. In contrast, we didn’t detect any positive blue staining particles within tendons in the other three groups. These results revealed the potential of PLGA/IO MPs for the long-term labeling of TSCs in vivo. Both MR and PA imaging had the capability of monitoring TSCs for at least 21 days and 7 days respectively in the rat rotator cuff injury model.

### Effect of TSCs transplantation on rotator cuff injury in rats

**Figure 9:**
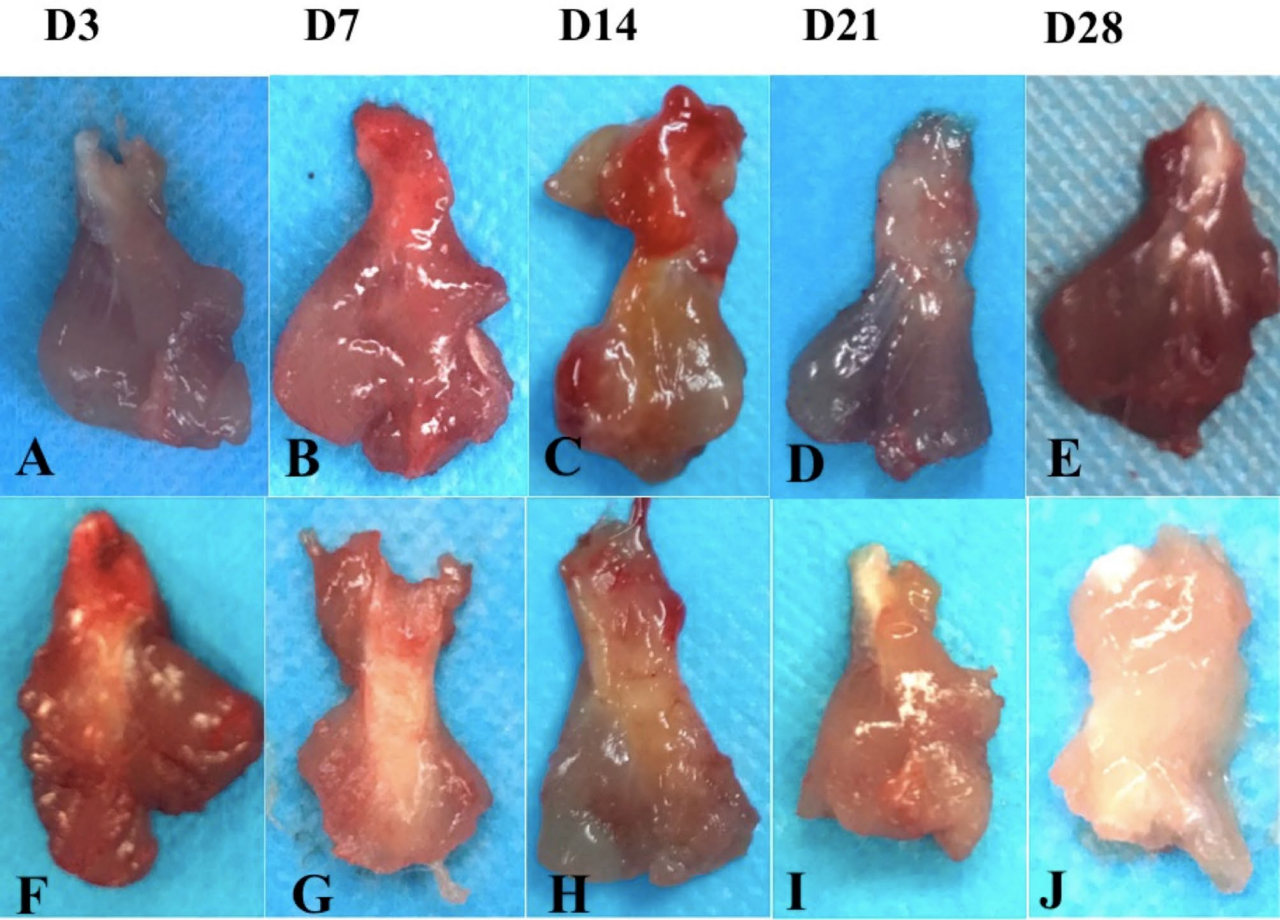
Visual observation of injured supraspinatus tendon. Appearance of tendon in the labeled TSCs group (**A**–**E**) and the untreated group (**F**–**J**) was evaluated at day 3, 7, 14, 21, and 28.

To detect the effect of TSCs for treatment of rotator cuff injury in rats, we assessed the appearance and histochemical staining of supraspinatus tendon at 3, 7, 14, 21 and 28 days after transplantation. As shown in Fig. 9A, rats received either labeled TSCs or unlabeled TSCs embedded in fibrin sealant implantation (rats in the labeled TSCs group or in the unlabeled TSCs group) demonstrated a significant less bleeding and swelling than rats in the FS group and the untreated group at day 3. In addition, muscles and tendons were covered with translucent membrane at day 7 (Fig. 9B), and covered with a thicker layer of translucent tissue at day 14 which may indicate the occurrence of recovery^27^ in the two group that received TSCs transplantation (Fig. 9C). This coating had turned milky white and opaque at day 21 (Fig. 9D). Finally, the tendons were healed with an appearance similar to normal tendon at day 28 (Fig. 9E). While in the FS group and the untreated group, we didn’t observe any translucent tissue until day 14 (Fig. 9H), and the muscles and tendons had turned pale and delicate with poor elasticity at day 28 (Fig. 9J).

**Figure 10:**
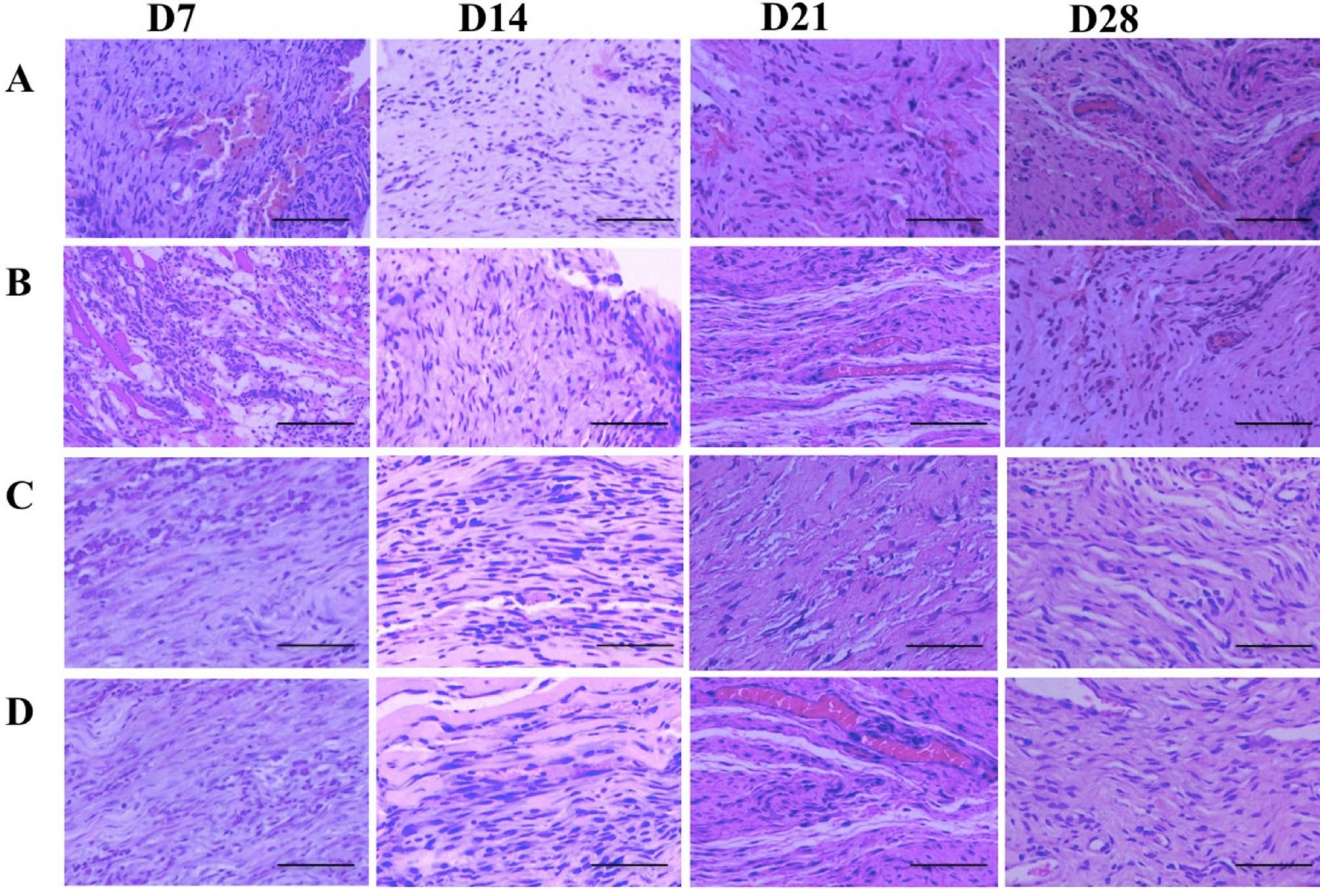
H&E staining of injured supraspinatus tendon at day 7, 14, 21 and 28 in the untreated group (**A**), FS group (**B**), labeled TSCs group (**C**) and unlabeled TSCs group. At day 7, more inflammatory cells were observed in the untreated group (**A**) and FS group (**B**) While rats in both labeled TSCs group (**C**) and unlabeled TSCs group (**D**) demonstrated more elongated fibroblasts at day 14. Scale bars = 100 μm.

The H&E and Masson staining indicated the disruption of tendon fibers in all the four groups at day 3 (Fig S4). There were more inflammatory cells within tendons in the untreated and FS group than that in the labeled TSCs group and unlabeled TSCs group at day 3 and 7 (Fig. 10). The two groups that received TSCs delivery demonstrated more elongated fibroblasts at day 14, increased extracellular matrix (ECM) formation at day 21, and more aligned cells parallel to the tendon longitudinal axis at day 28 (Fig. 10C,D). Cellularity in the FS group and untreated group was higher than the labeled TSCs group and unlabeled TSCs group at both time points.

**Figure 11:**
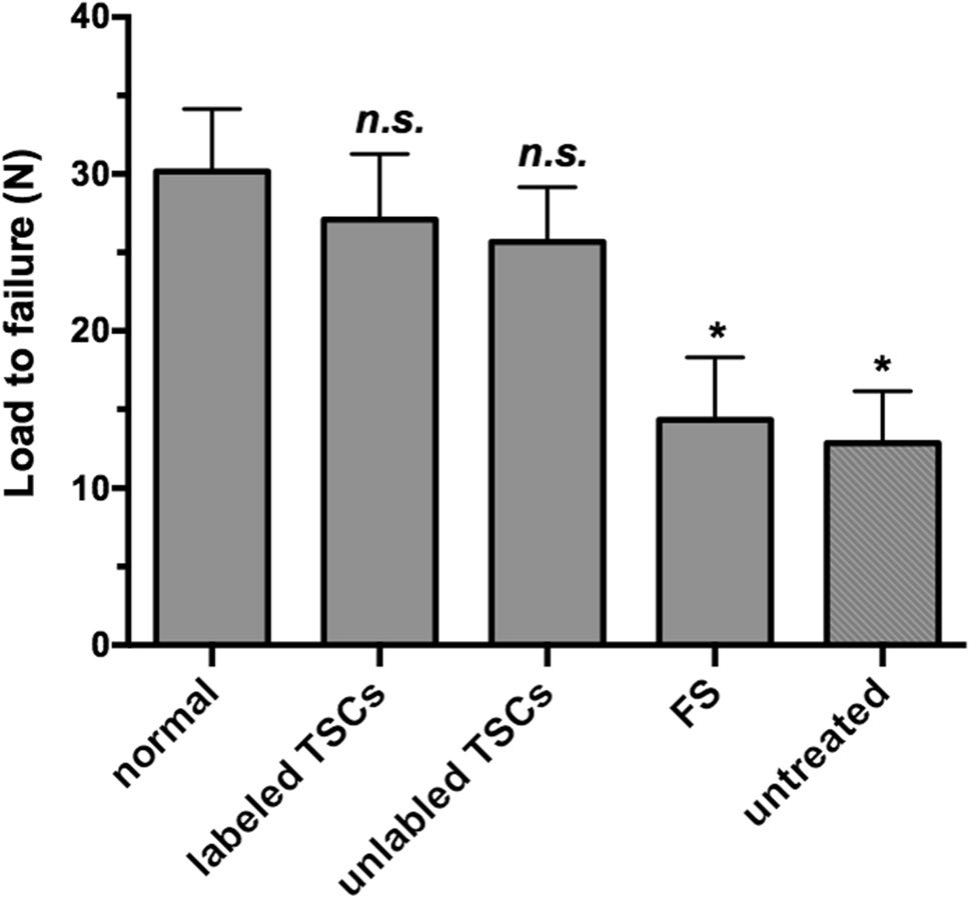
Biomechanical test of supraspinatus tendon in each group (n = 6). The mean load to failure of tendon in both labeled TSCs group and unlabeled TSCs was equal to that of normal tendon, and greater than that in FS group and untreated group. *, *P* < 0.05.

Finally, the biomechanical testing of supraspinatus tendons demonstrated that the mean load to failure in both labeled TSCs group and unlabeled TSCs group was equal to that of normal tendons (27.1 ± 4.20 N VS 30.2 ± 3.97 N, *P* > 0.05; 25.7 ± 3.50 N VS 30.2 ± 3.97 N), and was significantly greater than that of tendons in the untreated group (12.8 ± 3.31 N, *P* < 0.05) and the FS group (14.3 ± 3.98 N, *P* < 0.05) respectively (Fig. 11). The rats had achieved full restoration of injured supraspinatus tendon at 8 weeks after TSCs implantation. These results revealed the implantation of TSCs could improve and accelerate tendon healing in the rat rotator cuff model, and the therapeutic effect of TSCs was unaffected by PLGA/IO MPs labeling.

## Discussion

Our study demonstrated that (1) PLGA/IO MPs (PLL) has no adverse effect on cell viability and proliferation; (2) PLGA/IO MPs (PLL) was able to label TSCs and these labeled cells could be noninvasively monitored by both MRI and PA imaging for 21 days and 7 days respectively in rat rotator cuff injury model; (3) The therapeutic capacity of TSCs was not affected by PLGA/IO MPs labeling in a rat model of rotator cuff injury.

As we known, iron oxide nanoparticles (IO NPs) like superparamagnetic iron oxide (SPIO) have been extensively used as contrast agents for MR tracking because of their favorable biocompatibility and the high sensitivity in T2*-weighted images^17,28–31^. The sensitivity is dependent on the IO NPs loading of the cell as well as the density of labeled cells in an imaging voxel^32^. It is reported that positive surface charge particles are more easily endocytosed into cells and bigger particles are exocytosed at a slower rate^28^, which would lead to high loading of cells with tracers and increased cell labeling efficiencies. Xu et.al also demonstrated that confinement of IO-NPs in micron-sized PLGA particles (0.8 μm) leads to longer detectable time of labeled MSCs compared to IO-NPs (10 nm)^33^. Therefore, we fabricated a positive charged micron sized (about 1 μm) PLGA/IO particles to label TSCs in our study. Our previous study demonstrated that PLGA/IO MPs showed not only enhanced negative MR signal but also enhanced PA signal, and PLGA/IO MPs labeled TSCs allowed to be detected by dual-modal MR/PA imaging^25^.

In this study, we further detect the efficiency of PLGA/IO MPs for noninvasive longitudinal tracking TSCs via dual-modal MR/PA imaging both in vitro and in vivo. The in vitro experimental showed that 100 μg Fe/mL PLGA/IO MPs (PLL) labeling TSCs (for 12 h) was biological safe to label TSCs. ICP-OES quantification results revealed the efficient loading of PLGA/IO MPs for 21 days, which ensures the longitudinal tracking of TSCs by MRI and PA. Labeled TSCs suffered from time-dependent decrease in MR and PA signal due to cell proliferation and exocytosis of particles. MRI and PA allowed a long-term tracking of labeled TSCs for 21 days and 14 days, and allowed the detection limit of 5 × 10^4^ cells/mL, 1 × 10^5^ cells/mL respectively in vitro. While, labeled TSCs transplanted in the rat rotator cuff were capable of being visualized for 21 days and 7 days using MRI and PA imaging. These results indicate the ability of PLGA/IO MPs for noninvasive monitoring of TSCs via dual-modal MR/PA imaging. But the sensitivity of PA for tracking PLGA/IO MPs labeled TSCs was lower than that of MRI. It may suggest that PLGA/IO MPs has greater efficiency of monitoring TSCs via MRI than that via PA imaging.

Photoacoustic imaging is a compound imaging technology including optical imaging and US imaging based on photoacoustic effect. The photoacoustic effect is the generation of acoustic waves due to thermal expansion following the absorption of light^16^. Carbon nanotubes (CNTs), gold NPs, graphene and various organic dyes including indo-cyanine green (ICG) have been extensively studied as PA contrast agents due to their strong plasmon resonance (SPR) peaks in the near infrared region (NIR)^34^. Nam et.al applied gold nanotracers (Au NT) to label MSCs and demonstrated that US/PA imaging allowed a high detection sensitivity of 1 × 10^4^ cells/mL labeled MSCs and a longitudinal cell tracking for over one week time period^35^. Kim et al. applied prussian blue nanoparticles (PB NPs) to label MSCs and found that PA allowed the detection and monitoring of 5 × 10^4^ mesenchymal stromal cellsin living mice over a period of 14 days^16^. Like Au NPs and PB NPs, IO NPs or nanocomposites that contain IO NPs, also have the ability to exhibit a photothermal effect^24,25^. Sivakumar also reported the use of SPION encapsulated PLGA NPs as an alternative PA contrast agent and found that the pancreatic cancer cells treated with the nano-composite were ablated by the NIR laser at 800 nm^36^. Moreover, IO NPs or IO nanocomposites have strong magnetic property and incomparable biocompatibility, which make them promising dual-modal MR/PA contrast agents.

TSCs being a tendon native cell population, hold great promise for treatment of tendon injury^37,38^. Fibrin sealant is a two-component material consisting of fibrinogen and thrombin which is the only agent presently approved as a hemostat, sealant, and adhesive by the Food and Drug Administration (FDA)^39^. Here, we used it as a carrier to delivery TSCs, because that thrombin converts fibrinogen into insoluble fibrin that could prevent the leakage of TSCs suspension from the site of transplantation. Following delivery of TSCs, results from pathology and biomechanical test showed significant recovery in the supraspinatus tendon. Moreover, no difference in therapeutic effect including the appearance, histology and biomechanical test of supraspinatus tendon was observed between labeled TSCs group and unlabeled TSCs group. This indicated that the labeling of TSCs with PLGA/IO MPs may not alter the therapeutic outcome of TSCs. Similarly, Jokerst had synthetized silica coated gold nanorods to label MSCs and suggested that the therapeutic benefit of the MSCs will be retained after labeling^40^. Unfortunately, the cellular and molecular mechanisms leading to the improvement of tendon healing were still not clear. The therapeutic effect of TSCs may not be related to cell replacement, but rather from paracrine effects. Komatsu introduced TSCs sheets into a rat Achilles tendon defect and suggested that TSC may promote tendon repair probably by modulating the early inflammatory response and producing trophic factors that regulate the extracellular matrix^41^.

In conclusion, we have synthesized a novel PLGA/IO MPs, which was proved to be efficient for longitudinal labeling TSCs via both MRI and PA imaging. The sensitivity of MRI was greater than that of PA for tracking PLGA/IO MPs labeled TSCs. Moreover, TSCs implantation improved the repair of injured tendon that was not affected by PLGA/IO MPs labeling in the rat rotator cuff injury model. Our study indicated that PLGA/IO particle was a promising dual-modal MR/PA contrast for noninvasive long-term stem cell tracking.

## Supplementary information

Supplementary Information.
