# Supplementary material for: Dual-modal magnetic resonance and photoacoustic tracking and outcome of transplanted tendon stem cells in the rat rotator cuff injury model
Source: Sci Rep. 2020 Aug 18;10:13954. doi: 10.1038/s41598-020-69214-5 (PMC7435193; doi:10.1038/s41598-020-69214-5)
Supplement: Supplementary file 1 — Supplementary Information. [file 41598_2020_69214_MOESM1_ESM.pdf]

Dual-modal Magnetic Resonance and Photoacoustic Tracking and Outcome of Transplanted Tendon Stem Cells in the Rat Rotator Cuff Injury Model

Xueqing Cheng[1*], Jinshun Xu[2*], Ziyue Hu[1,3], Jingzhen Jiang[1,3], Zhigang Wang[4], Man Lu[1#]

The isolation, culture and identification of rat TSCs

TSCs were harvested from 6 ~ 8 weeks old male SD rats based on the previous protocol described by Bi et.al1. Following anesthesia, the bilateral achilles tendons were acquired and cut into small pieces (1mm×1 mm), then digested with 3mg/ml collagenase type I (Sigma, C0130-1G) and 4mg/ml dispase (Sigma, D4693-1G) in PBS for 2 h at 37°C. The isolated cell suspensions were primary cultured and subcultured in DMEM (Gibco), supplemented with 10% (v/v) FBS (Hyclone) and 1% (v/v) penicillin/ streptomycin (Beyotime) at 37 °C, 5% CO_2. Cells were cultured to 70~80% confluence before passaging. All experiments were performed using P3 ~P7 TSCs. Before cell experiments, TSCs were detached with Trypsin 0.05%-EDTA 0.53mM(Gibco).

Preparation of PLGA/IO MPs

PLGA/IO MPs were prepared using double emulsion method. Briefly, 200ul Fe_3O_4 NPs (10 nm, 25mg/ml, Ocean Nanotech, AR) coated with oleic acid, 200ul deionized water (H_2O) were mixed with 30 mg PLGA (lactide: glycolide =50:50, MW=10000Da, Daigang Biomaterial Co., Ltd, Jinan, ShanDong) in 1 mL chloroform, then the solution was sonicated for 1 min at 125w using acoustic vibration (VCY-500, Shanghai, China) to achieve an emulsion in a 50 mL beaker. Subsequently, 10ml 4% PVA solution was added and dispersed by a high-shear dispersion homogenizer at B level (HENC) for 2 min to form the second emulsion. The homogenized mixture was then stirred for 4 h in a chemical fume hood at room temperature to evaporate

chloroform. Finally, particles were isolated by centrifugation at 6,000 rpm for 5 minutes, washed twice with deionized water, and then redispersed in 1.5ml deionized water and stored at 4°C for standby application.

References

1. Bi, Y.; Ehirchiou, D.; Kilts, T. M.; Inkson, C. A.; Embree, M. C.; Sonoyama, W.; Li, L.; Leet, A. I.; Seo, B. M.; Zhang, L.; Shi, S.; Young, M. F., Identification of tendon stem/progenitor cells and the role of the extracellular matrix in their niche. Nat Med 2007, 13 (10), 1219-27.

Figures

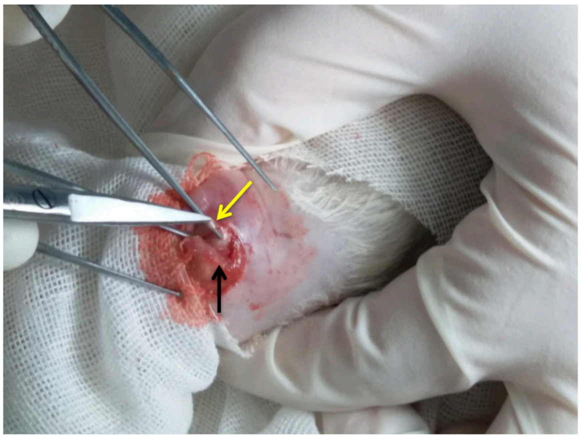

Figure S1. Visualization of supraspinatus tendon (yellow arrow) of rat after splitting deltoid (black arrow)

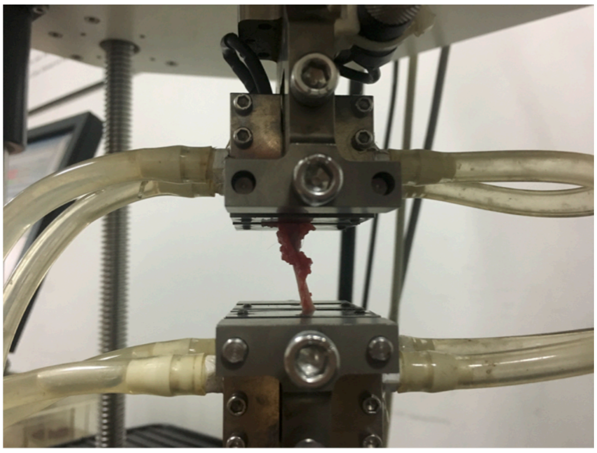

Figure S2. The supraspinatus tendon was fixed to the electrodynamic test machine

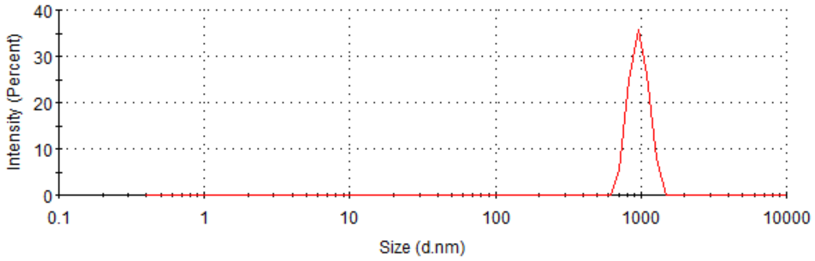

A

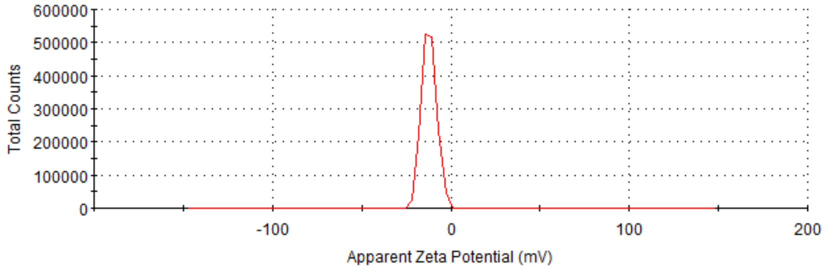

B

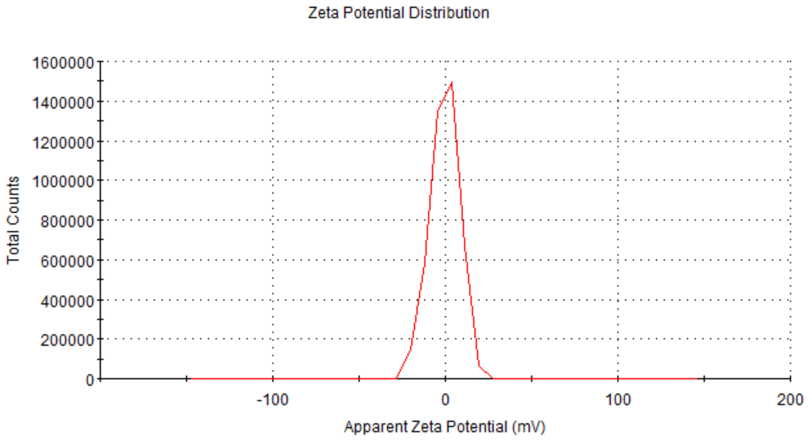

C

Figure S3. (A) The average particle size of PLGA/IO MPs. (B) The zeta potential of PLGA/IO MPs. (C) The zeta potential of PLGA/IO MPs after coating with PLL.

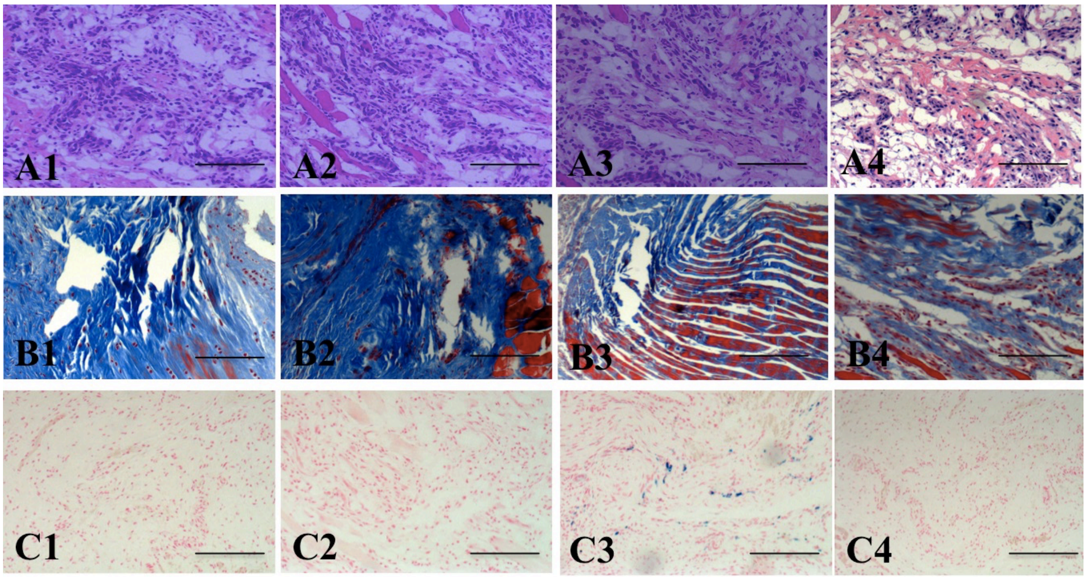

Figure S4. Histological analysis of the supraspinatus tendon in the untreated group (A1~C1), FS group (A2~C2), labeled TSCs group (A3~C3) and unlabeled TSCs at day 3. (A) H&E staining showed less inflammatory cells in the labeled TSCs (A3) and unlabeled TSCs group (A4) compared with the other two groups (A1 and A2). (B) Masson's trichrome staining showed the disruption of tendon fibers (blue-stained) in each group at day 3. Scale bar as represented. (C) Prussian blue staining detected blue-staining particles only in the labeled TSCs group (C3).

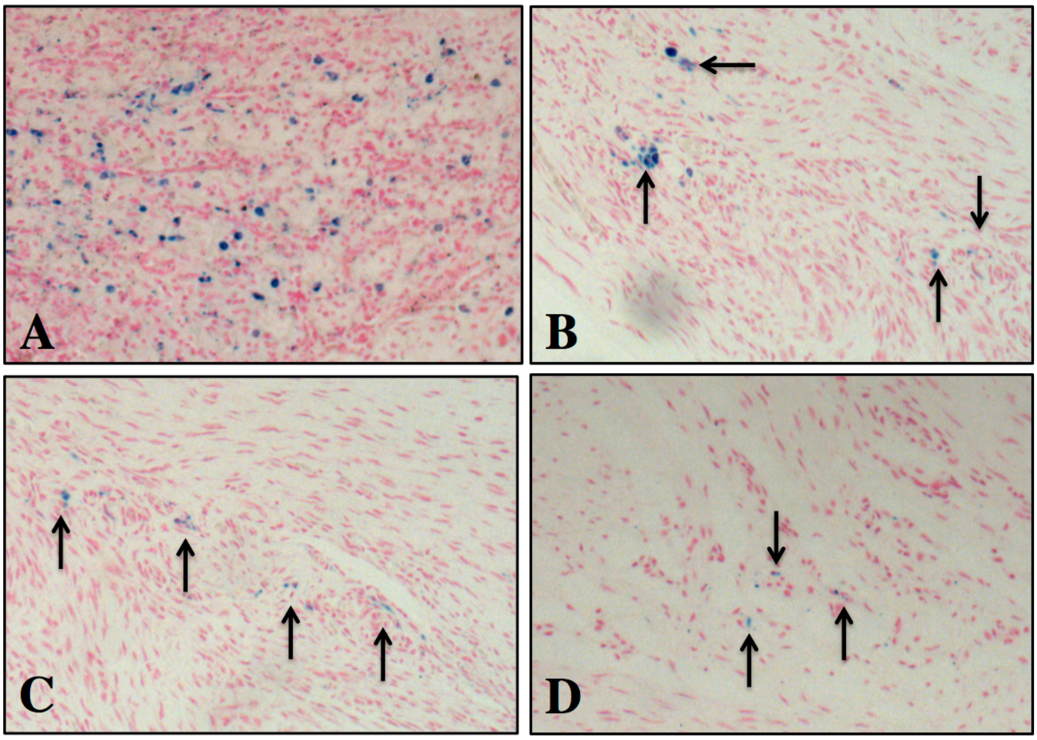

Figure S5. Prussian blue staining of injured tendon in the labeled TSCs group at day 7 (A), 14 (B), 21 (C) and 28 (D). Blue-staining particles (arrows) decreased on the tissue section of tendon over time.
